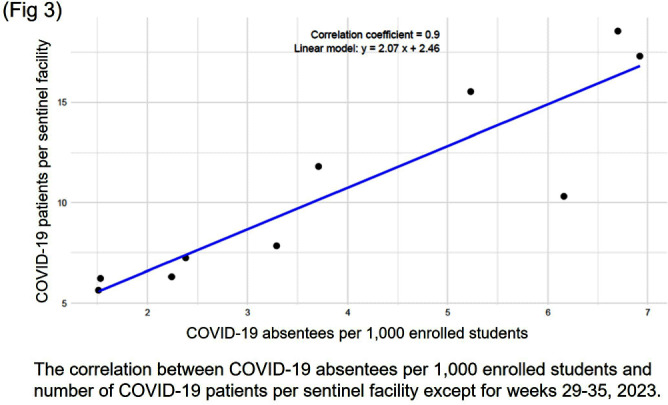# Comparison of COVID-19 Sentinel Surveillance and COVID-19 School Absentee Surveillance in Japan

**DOI:** 10.1017/ash.2024.325

**Published:** 2024-09-16

**Authors:** Masaki Tanabe, Shiho Ito, Yasuyuki Hara, Takahiro Ogura, Makoto Ichikawa, Miwa Fukuta, Yoshito Iwade

**Affiliations:** Mie University Hospital; Department of Medical Health, Mie Prefectural Government, Mie, Japan; Mie Prefectural Institute of Public Health and Environmental Sciences

## Abstract

**Background:** In Japan, notifiable infectious disease surveillance ended and was replaced by sentinel surveillance following the COVID-19 reclassification in May 2023. Since COVID-19 sentinel surveillance is integrated into seasonal influenza surveillance, the number of reported cases varies depending on the extent to which sentinel facilities provide COVID-19 care. Therefore, we compared COVID-19 sentinel surveillance with school absentee surveillance, which is limited to high school equivalent age or younger, but provides reliable information on absences in the target population. **Method:** The 17-week period from week 23 (June 5 to June 11) to week 39 (September 25 to October 1) of 2023 was used as the target period. The number of weekly COVID-19 reports from 72 sentinel sites in Mie Prefecture (Population 1.7 million) as for sentinel surveillance and the number of COVID-19 absentees at a total of 998 facilities (401 kindergartens and nursery schools, and 597 elementary, junior high, and senior high schools) registered for school absentee surveillance in Mie Prefecture as for school absentee surveillance were compared across Mie Prefecture and eight health centers (Fig 1). **Result:** Except for the summer vacation period from week 29 to 35, sentinel surveillance and school absentee surveillance showed a significant positive correlation. During the summer vacation period, a decrease in the number of COVID-19 absentees was observed, especially in the elementary, junior high, and senior high school groups of the school absentee surveillance, compared to the sentinel surveillance (Fig 2 and 3). When compared by health center, no regional differences were observed in school absentee surveillance, but in sentinel surveillance, some health centers reported significantly more cases than others. **Conclusion:** The results of this study suggest that although COVID-19-based school absentee surveillance has some drawbacks, such as the limited number of subjects and the difficulty of evaluation during the summer vacation when schools are closed, it has the advantage of being able to evaluate the entire community without being affected by medical institution practice bias, and can be used to monitor trends in infectious diseases. It was considered important to combine and evaluate multiple surveillance indicators in order to accurately monitor epidemiologic trends of infectious disease over time.

**Figure:**
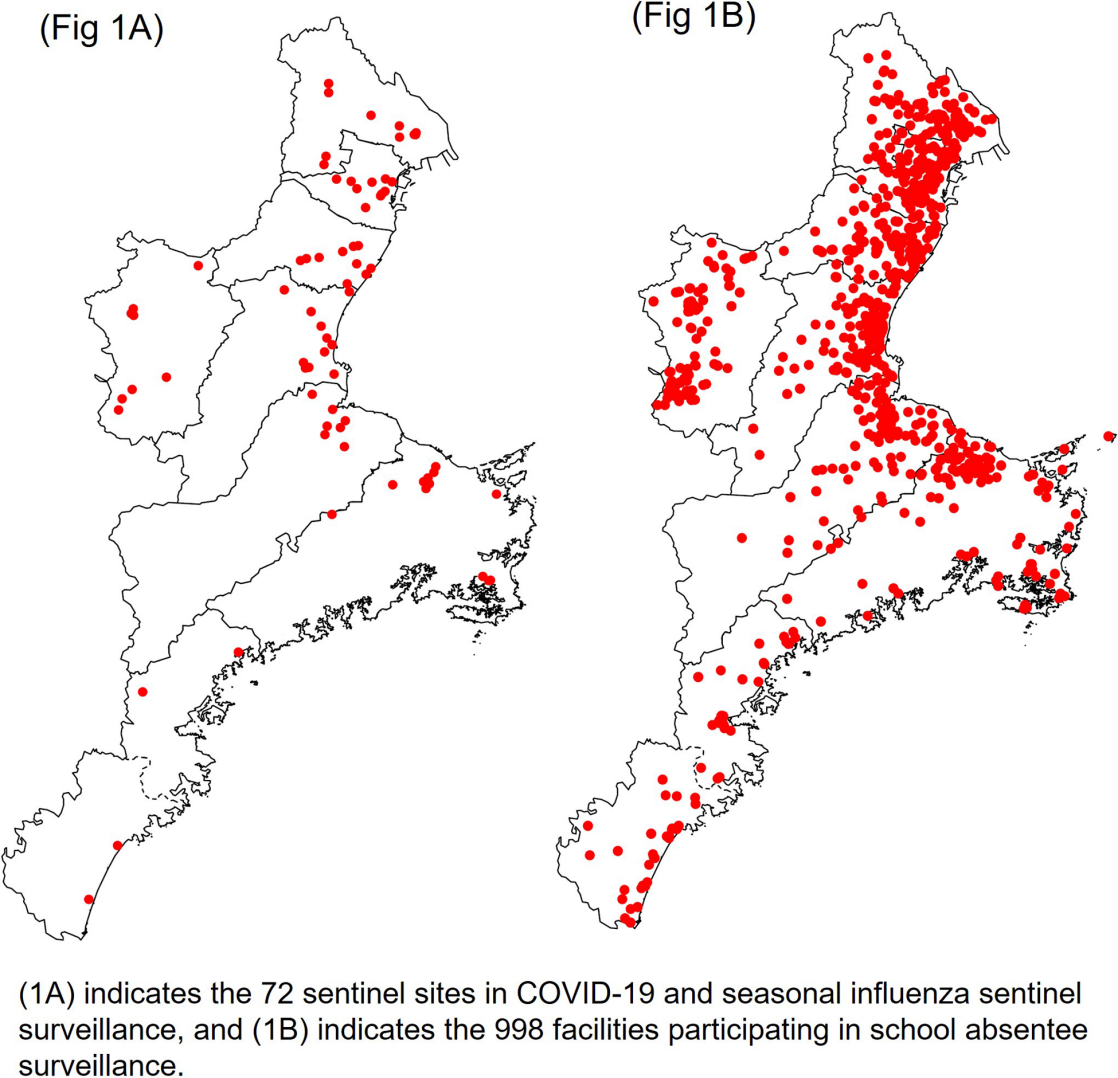

**Figure:**
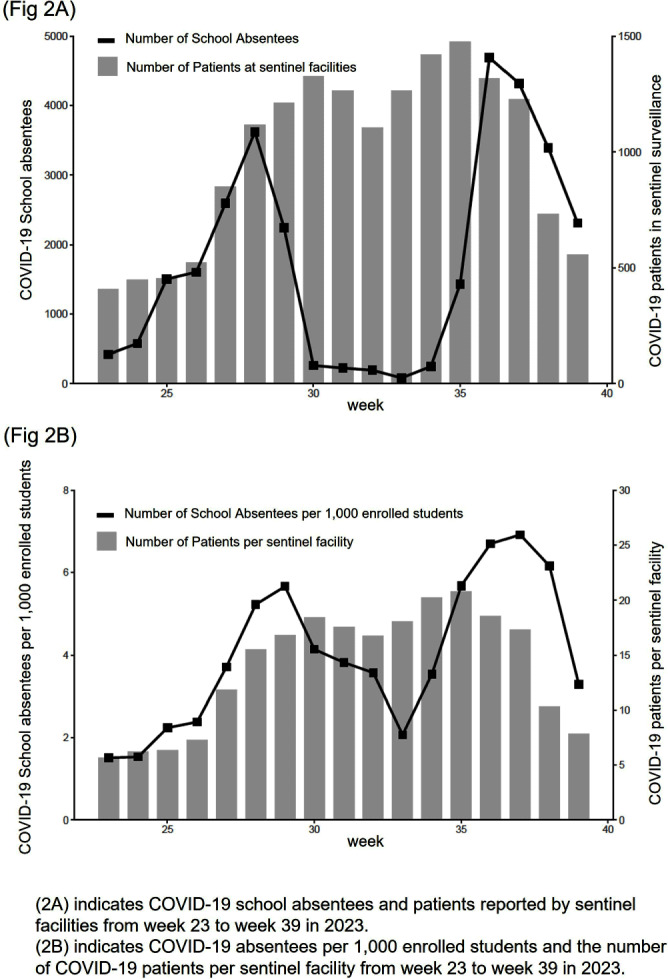

**Figure:**